# Anti-EGFR bioengineered bacterial outer membrane vesicles as targeted immunotherapy candidate in triple-negative breast tumor murine model

**DOI:** 10.1038/s41598-023-43762-y

**Published:** 2023-09-29

**Authors:** Razieh Rezaei Adriani, Seyed Latif Mousavi Gargari, Hamid Bakherad, Jafar Amani

**Affiliations:** 1 Department of Biology, Shahed University, Tehran, Iran; 2 Department of Pharmaceutical Biotechnology, Isfahan University of Medical Sciences, Isfahan, Iran; 3 Applied Microbiology Research Center, System Biology, and Poisonings Institute, Baqiyatallah University of Medical Sciences, Tehran, Iran

**Keywords:** Cancer, Drug discovery

## Abstract

Cancer immunotherapy employing checkpoint inhibitors holds great promise across diverse cancers; nonetheless, a substantial proportion of patients (ranging from 55 to 87%) remain unresponsive to this treatment. To amplify therapeutic efficiency, we propose a synergistic therapeutic strategy that entails the deployment of targeted nano-sized particles carrying Toll-like receptor (TLR) agonists to the tumor site. This innovative approach seeks to activate intratumoral antigen-presenting cells using bioengineered outer membrane vesicles (OMVs) derived from gram-negative bacteria. These OMVs possess inherent attributes of surface-exposed immune stimulators and TLR-activating components, rendering them intriguing candidates for investigation. These OMVs were meticulously designed to selectively target cancer cells exhibiting an overexpression of epidermal growth factor receptor (EGFR). To gauge the precision of this targeting, the conducted affinity-based assays aimed at determining the equilibrium dissociation constant of the single-chain variable fragment employed for this purpose. In vitro experiments confirmed the OMVs' proficiency in adhering to EGFR-overexpressed cancer cells. Moreover, the evaluation extended to an in vivo context, where the therapeutic effect of nanovesicles was appraised within the tumor microenvironment of the triple-negative breast cancer mouse model. Notably, both intraperitoneal and intratumoral administrations of nanovesicles exhibited the ability to activate natural killer cells and skew M2 macrophage towards an M1 phenotype. The combined scrutiny of in vitro and in vivo findings underscores the potential efficiency of OMVs as a promising strategy for future anti-tumor endeavors.

## Introduction

Cancer immunotherapy, including chimeric antigen-receptor therapies and immune checkpoint blockade, has emerged as a promising approach in the treatment of various cancers. Despite its success in some cases, a significant proportion of patients (approximately 55–87%) do not respond favorably to checkpoint inhibitors. To improve response rates, combinatorial therapy has gained attention, focusing on innate immune agonists such as Toll-like receptors (TLRs) and stimulators of interferon genes (STING agonists). This approach aims to activate intratumoral antigen-presenting cells (APCs) and facilitate the presentation of tumor antigens to effector T cells, thereby enhancing the effectiveness of immunotherapy^1^. Although many researchers have focused on the targeted delivery of chemotherapy agents using nanoparticles, nano-immunotherapy has been minimally investigated. Targeted nano-size particles (NPs) can be engineered to deliver immunotherapeutic agents in their native conformation to the tumor site and enhance the therapeutic index by direct delivery of immunotherapeutic agents to the site of interest, leading to the accumulation and potency at the tumor region and minimizing the dose-dependent systemic toxicity. On the other hand, NPs can easily pass through the blood and lymphatic vessel barriers and interact with and stimulate immune cells^2^. Outer membrane vesicles (OMVs) are lipid-bilayer vesicles secreted by all gram-negative bacteria. These vesicles possess a unique characteristic of presenting diverse surface-exposed immune stimulators in their native conformation, along with components that activate Toll-like receptors (TLRs)^3,4^. Leveraging this natural property, OMVs hold great potential in cancer immunotherapy, as they have the ability to induce both humoral and cell-mediated immune responses, thereby enhancing the immune system's ability to target cancer cells^4^. Acellular bacterial OMVs have gained recognition as valuable delivery vehicles, particularly in the fields of cancer drug delivery and candidate vaccine development. However, concerns about OMV toxicity must be addressed when considering their use as cancer immunotherapy agents. To mitigate this concern, the application of detergents in OMV purification has been commercialized, resulting in efficient production and reduced lipopolysaccharide (LPS) toxicity^5,6^. Notably, OMVs have already demonstrated their potential in the clinical setting. For instance, an OMV-based vaccine targeting meningococcal group B has been successfully developed and is available for clinical use under the trade name Bexsero, showing their applicability in vaccine development^7^. Additionally, nanoparticles (NPs) can serve as an effective anti-immunosuppressive delivery system, delivering agents such as TGF-β receptor inhibitors directly to the tumor microenvironment. This approach enhances the activation of the immune system within the tumor region, presenting an innovative strategy to counteract immunosuppression and bolster the immune response against cancer^8^. The epidermal growth factor receptor (EGFR) plays a crucial role as a therapeutic target in various cancer types. Notably, besides being overexpressed in certain solid tumors like head/neck and colorectal cancer^9^, EGFR gene rearrangement (EGFRvIII) occurs in multiple cancer types. EGFRvIII expression is specifically observed in breast carcinoma, non-small cell lung carcinoma, and some high-grade glioma cases, making it an ideal candidate for immunotherapy^10^. Several monoclonal antibodies have been approved to target domain III of EGFR, including cetuximab, panitumumab, necitumumab, nimotuzumab, and matuzumab. Among these, only panitumumab possesses the unique ability to neutralize both wild-type EGFR and EGFRvIII, resulting in a reduction of p-S6 signaling and displaying superior in vitro and in vivo antitumor activity^11,12^. These findings highlight the potential of panitumumab as a valuable therapeutic agent in cancer treatment, particularly in cases involving EGFR and EGFRvIII. In this study, we utilized ClyA protein to display a high-affinity anti-EGFR ligand on the surface of OMVs. In fact, the scFv structure originated from panitumumab monoclonal antibody was expressed on the surface of bacteria as a fusion with ClyA to interact with highly overexpressing EGFR cancer cells in vitro. To confirm the target specificity of these engineered OMVs toward EGFR, we conducted affinity-based assays using both EGFR-positive and EGFR-negative cells, and subsequently calculated the equilibrium dissociation constant (Kd) of the designed scFv. The bioinformatic results further confirmed the high affinity of scFv for the EGFR^13^. Moving to in vivo experiments, we employed a syngeneic model of triple-negative mouse breast cancer (4T1), known for its challenges in terms of targeting and immunotherapy. Using this model, we evaluated the functional performance of bioengineered OMVs in vivo. Specifically, we investigated the combinatorial effects of these bioengineered OMVs, which involved EGFR inhibition and localized immune activation, and examined their impact on tumor growth. This approach aimed to explore the potential synergistic effects of both EGFR targeting and immune system activation in combating tumor growth within the challenging triple-negative breast cancer model.

## Results

### Surface displayed scFv-OMVs isolation and purification

**Figure 1:**
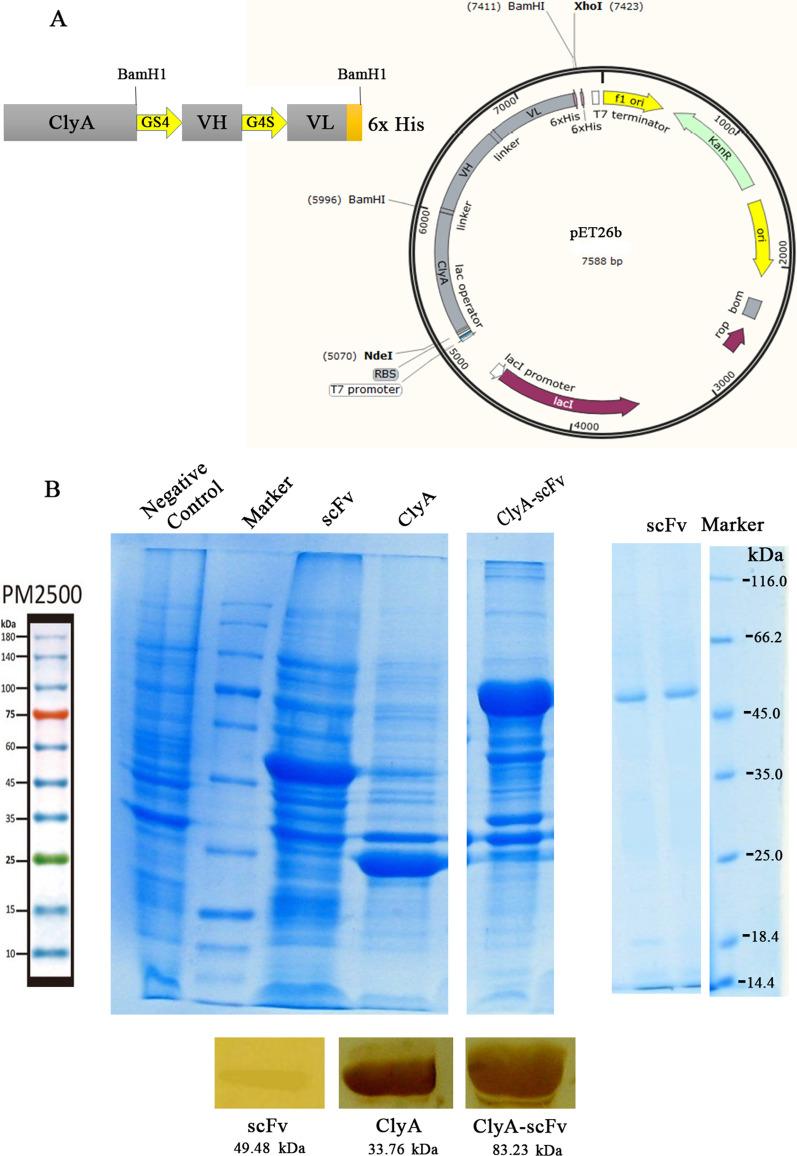
Protein expression analysis. (**A**) Schematic representation of ClyA-scFv construct, illustrating the arrangement of constituent fragments within the construct; (**B**) Protein expression analysis of ClyA-scFv, ClyA, and scFv in *E. coli* BL21 strain using 12% SDS-PAGE and Western blotting applied to total bacterial protein samples (left); The purified scFv protein expressed within the BL21 strain was subjected to 12% SDS-PAGE analysis (right).

**Figure 2:**
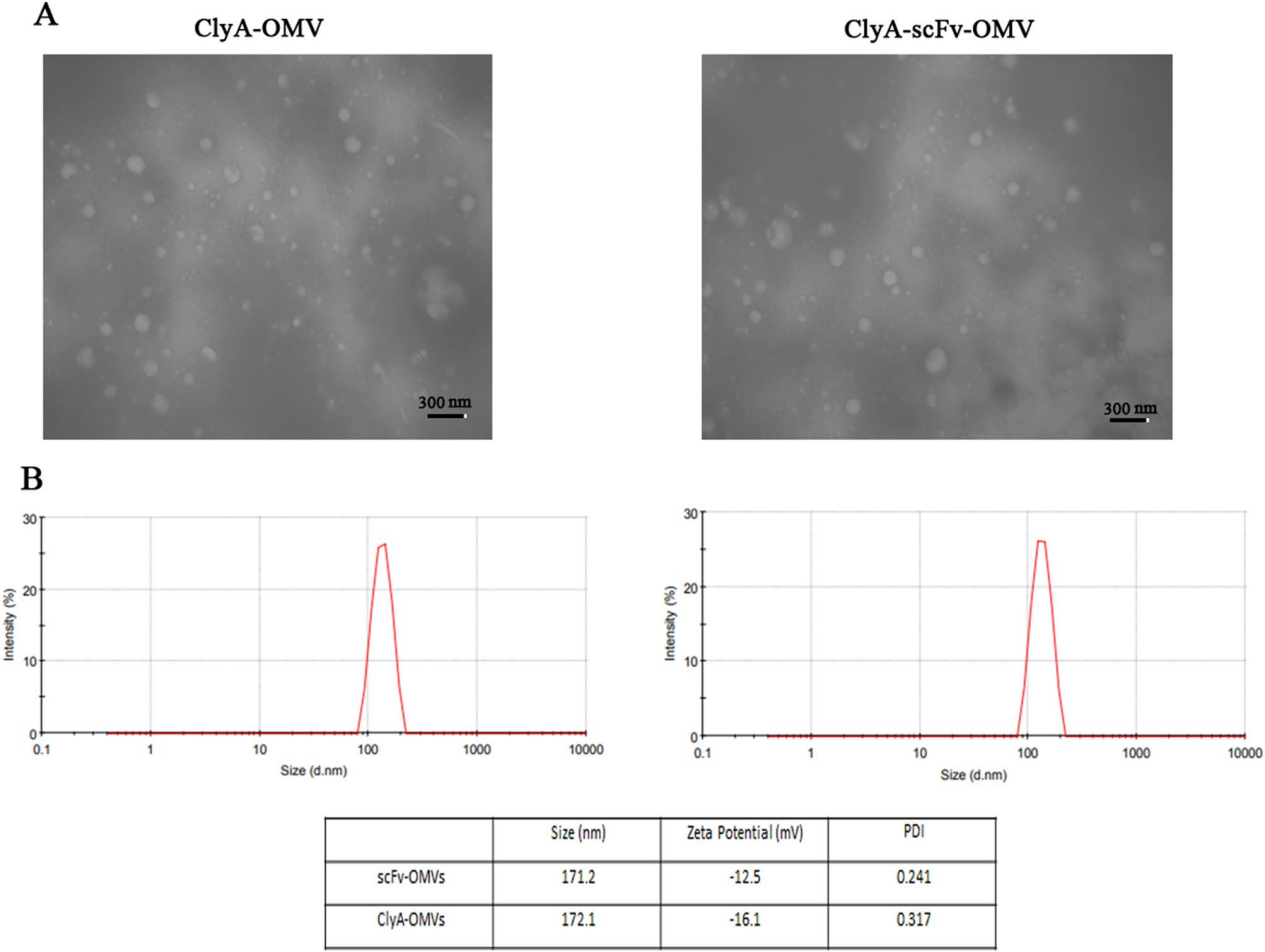
OMVs characterization. (**A**) TEM images depicting ClyA-OMVs and scFv-OMVs; (**B**) The zeta potential values of scFv-OMVs and ClyA-OMVs were determined as −12.5 and −16.1 mV, respectively. The scale bar corresponds to 300 nm.

In this investigation, outer membrane vesicles (OMVs) were derived from a non-pathogenic *E. coli* BL21 (DE3) strain using deoxycholate detergent to decrease the lipopolysaccharide (LPS) content within the vesicle structure. The ClyA-scFv construct (Fig. 1A) was introduced into these bacteria via transformation with the pET26b vector, aiming to produce scFv-OMVs with minimal cytotoxic effects. Induction of ClyA-scFv expression on the bacterial outer membrane was achieved through the addition of IPTG. Subsequent to bacterial treatment with detergent and several rounds of centrifugation and filtration, purification of scFv-OMVs was carried out. The expression of ClyA-scFv, along with ClyA and scFv used as negative and positive controls, was verified through SDS-PAGE and Western blot analysis employing HRP-conjugated anti-His tag antibody (Fig. 1B). The protein concentrations of scFv-OMVs, ClyA-OMVs, and scFv were measured at 8860 µg mL^−1^, 9326 µg mL^−1^, and 1225 µg mL^−1^, respectively. Additionally, transmission electron microscopy imaging demonstrated that both ClyA-OMVs and scFv-OMVs exhibited a uniform spherical shape (Fig. 2A), while dynamic light scattering (DLS) results revealed that the average diameters of ClyA-OMVs and scFv-OMVs were 172.1 and 171.2 nm, respectively (Fig. 2B). For the validation of purified OMVs sterility, a meticulous procedure was employed. Both ClyA-OMVs and scFv-OMVs were subjected to plating on LB agar followed by an incubation period of 24 h at 37 °C. The absence of any bacterial colonies on the agar plates confirmed the sterility of the OMVs preparation. Furthermore, to solidify the surface display of the ClyA-scFv structure on both bacteria and OMVs, a complementary analysis was performed utilizing the ELISA technique. Refer to Supplementary Fig. 7 for visual representation.

### scFv-OMVs exhibited specific binding to EGFR-overexpressing cells

**Figure 3:**
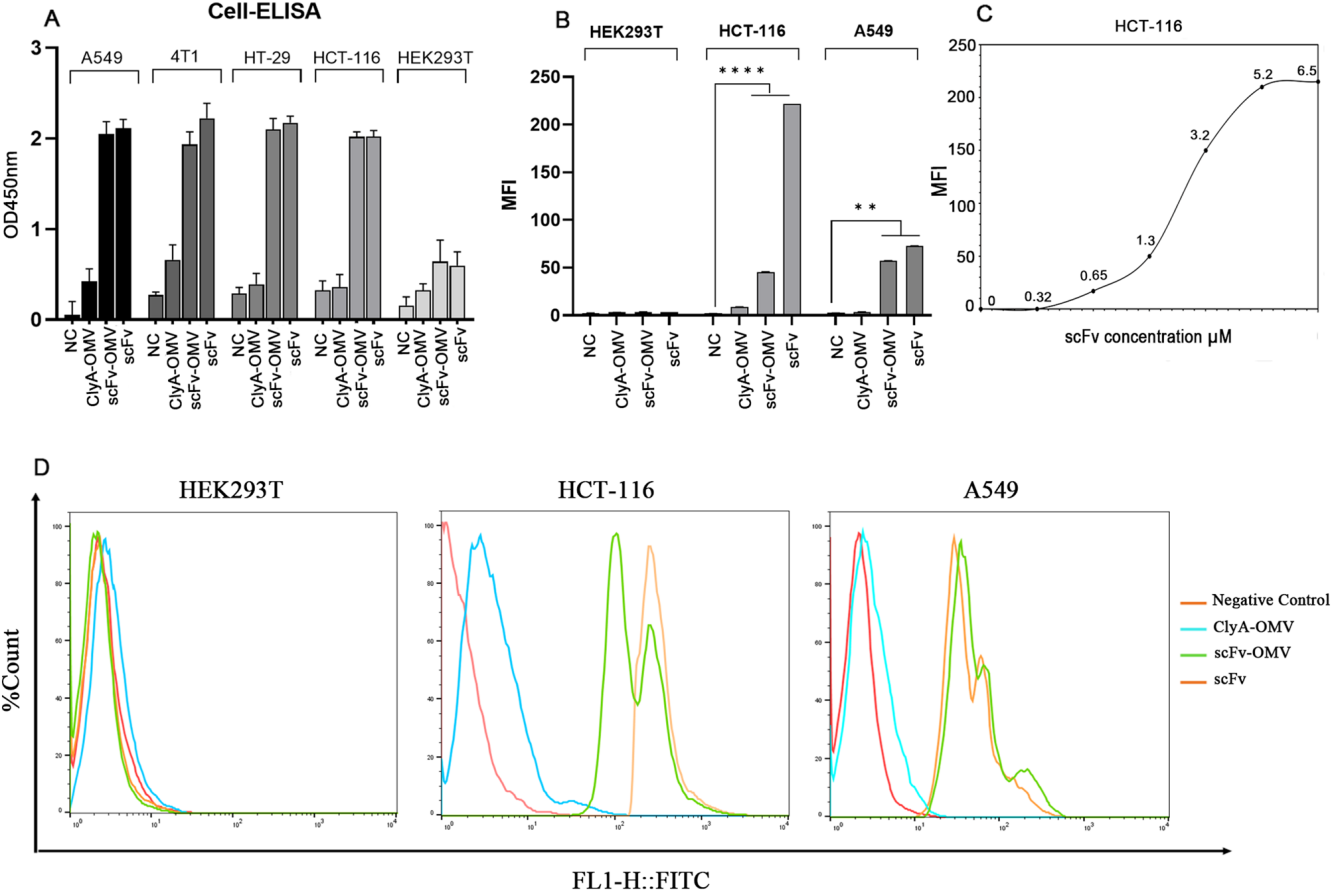
Binding capacity assessments of engineered OMVs against various EGFR high expression cell lines. (**A**) Cell ELISA assay was carried out using scFv-OMVs, ClyA-OMVs, scFv, and PBS as negative control (NC). Distinct binding was observed for scFv-OMVs and purified scFv against EGFR positive cell lines; (**B**) Mean fluorescent intensity (MFI) plot showcasing the interaction of scFv-OMVs, ClyA-OMVs, scFv protein, and FITC-conjugated IgG as negative control with HEK-293T, HCT-116, and A549 cell lines. Notably, ClyA-OMVs and FITC-conjugated IgG show no unspecific binding to the tumor cells. The data represented as mean ± SD (n = 3), were subjected to statistical analysis (**p < 0.05, ***p < 0.001 in comparison to negative control); (**C**) The equilibrium dissociation constant (Kd) of scFv-OMVs across various concentrations of surface displayed scFv protein (ranging from 0 to 6.5 µM) on OMVs. This evaluation was performed through interaction with 4 × 10^5^ of HCT-116 cells; (**D**) Flow cytometry analysis was employed to elucidate the binding of scFv-OMVs to EGFR-positive tumor cells. The shift in fluorescence signal evident in HCT-116 and A549 cell lines corresponds to the binding of scFv-OMVs and scFv protein to their respective receptor. The ClyA-OMVs and FITC-conjugated IgG were utilized as a negative control in this study.

To confirm whether the chimeric protein on the surface of OMVs maintains its affinity towards EGFR, a Cell-ELISA assay was conducted against EGFR overexpressing cells, namely HT-29, HCT-116, A-549, 4T1, and negative control, HEK-293T (Fig. 3). The binding characteristics of scFv-OMVs were compared to those of non-target OMVs (ClyA-OMVs) and purified scFv structure as a positive control. EGFR-overexpressing cell lines, including the triple-negative 4T1 cells, displayed significantly higher optical density when treated with both scFv-OMVs and the purified scFv structure, in comparison to non-EGFR expressing HEK-293T cell lines. Notably, ClyA-OMVs exhibited no binding to HEK-293T or other positive cell lines, indicating the absence of any nonspecific binding of the isolated OMVs to the investigated cell lines. To further investigate the binding capability of scFv-OMVs to EGFR, flow cytometry analysis was performed using HCT116 and A-549 cell lines. As observed in the results presented in Fig. 3D, the shift in fluorescent intensity for both scFv-OMVs and scFv correlates with the expression levels of EGFR on the surface of EGFR-positive cell lines. Accordingly, the HCT-116 cells demonstrated a more significant shift in fluorescent intensity compared to A-549 and the negative control HEK-293T cell lines, indicating a higher level of EGFR expression on the surface of HCT-116 cells. These findings align with the ELISA data, which also support the specific binding of scFv-OMVs to EGFR.

### Kd evaluation of scFv-EGFR interaction by flow cytometry

The binding affinity (Kd) of scFv-OMVs for EGF receptors was assessed through flow cytometry using HCT-116 cells. Different concentrations of scFv-OMVs were incubated with the cells, and the average fluorescent intensity was measured to determine the binding affinity. Figure 3 presents the Kd values of FITC-labeled scFv-OMVs at concentrations of 2.6 µmol L^−1^ when incubated with 4 × 10^5^ cells.

### scFv-OMVs bind EGFR-overexpressing A549 cells

**Figure 4:**
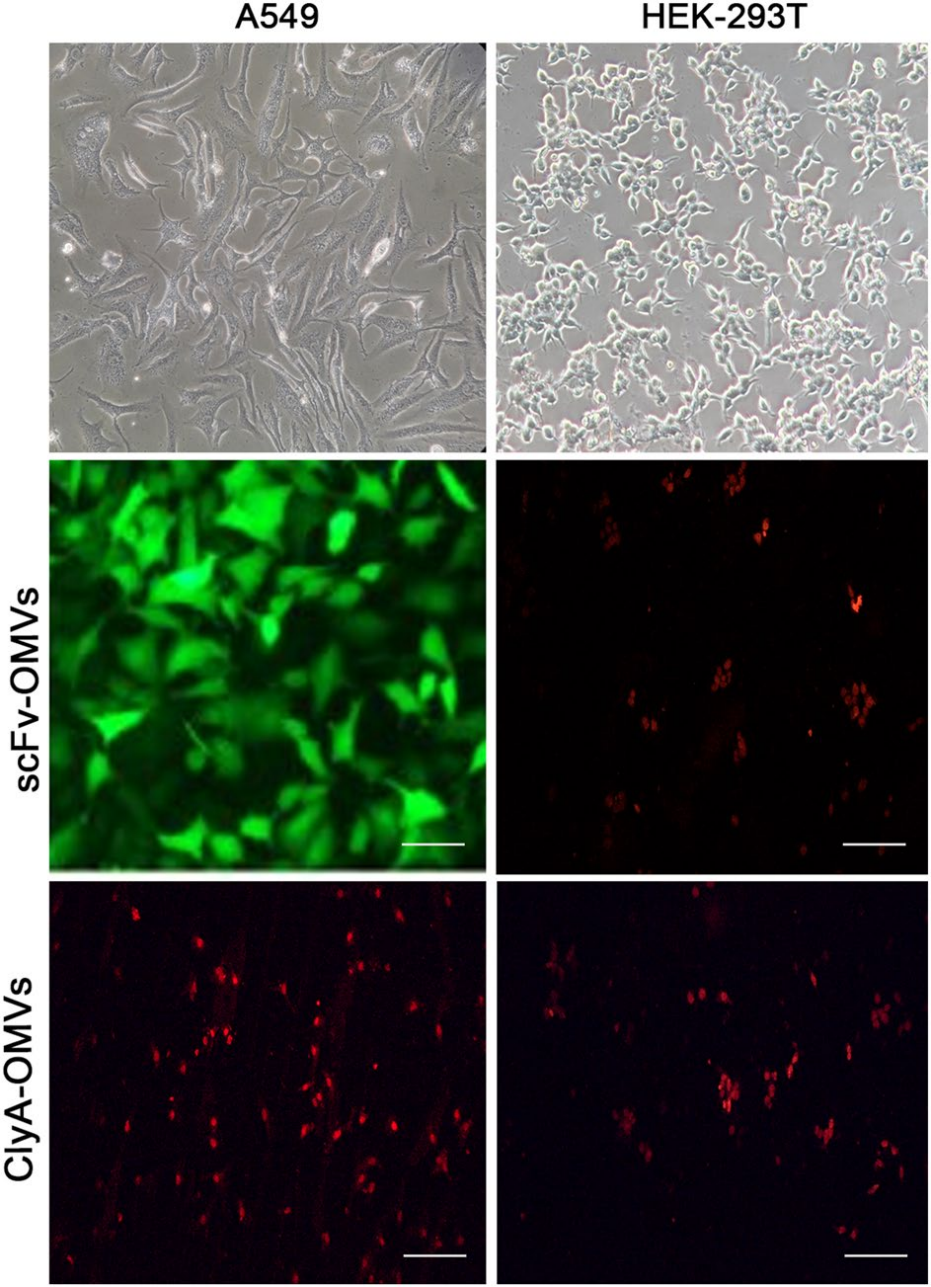
The binding analysis of scFv-OMVs by fluorescent microscopy. A549 and HEK-293T cells were incubated with scFv-OMVs and ClyA-OMVs for 1 h at room temperature. Following this incubation, no attachment was observed on A549 cells subjected to ClyA-OMVs incubation. Similarly, the HEK-293T cells did not display any binding of scFv-OMVs and ClyA-OMVs on their surface. For visualization, cells were stained with mouse anti-His tag antibody followed by FITC conjugated goat anti-mouse IgG. Scale bars represent 100 µm.

To further investigate the scFv-mediated cellular selectivity through EGFR binding, fluorescent microscopy imaging was employed. Formaldehyde-fixed A549 cells (EGFR-overexpressing) and EGFR-negative HEK-293T cells were treated with scFv-OMVs, and their surface localization was observed through fluorescent microscopy. The results clearly indicate the specific cellular binding of scFv-OMVs in A549 cell lines, while no fluorescence was observed in A549 cells treated with ClyA-OMVs nor in HEK-293T cells (Fig. 4). Collectively, the outcomes from Cell-ELISA and fluorescent microscopy imaging provide strong evidence that scFv-OMVs selectively and effectively target EGFR, confirming their potential as a targeted delivery system for EGFR-overexpressing cells.

### In vitro efficacy of scFv-OMVs

**Figure 5:**
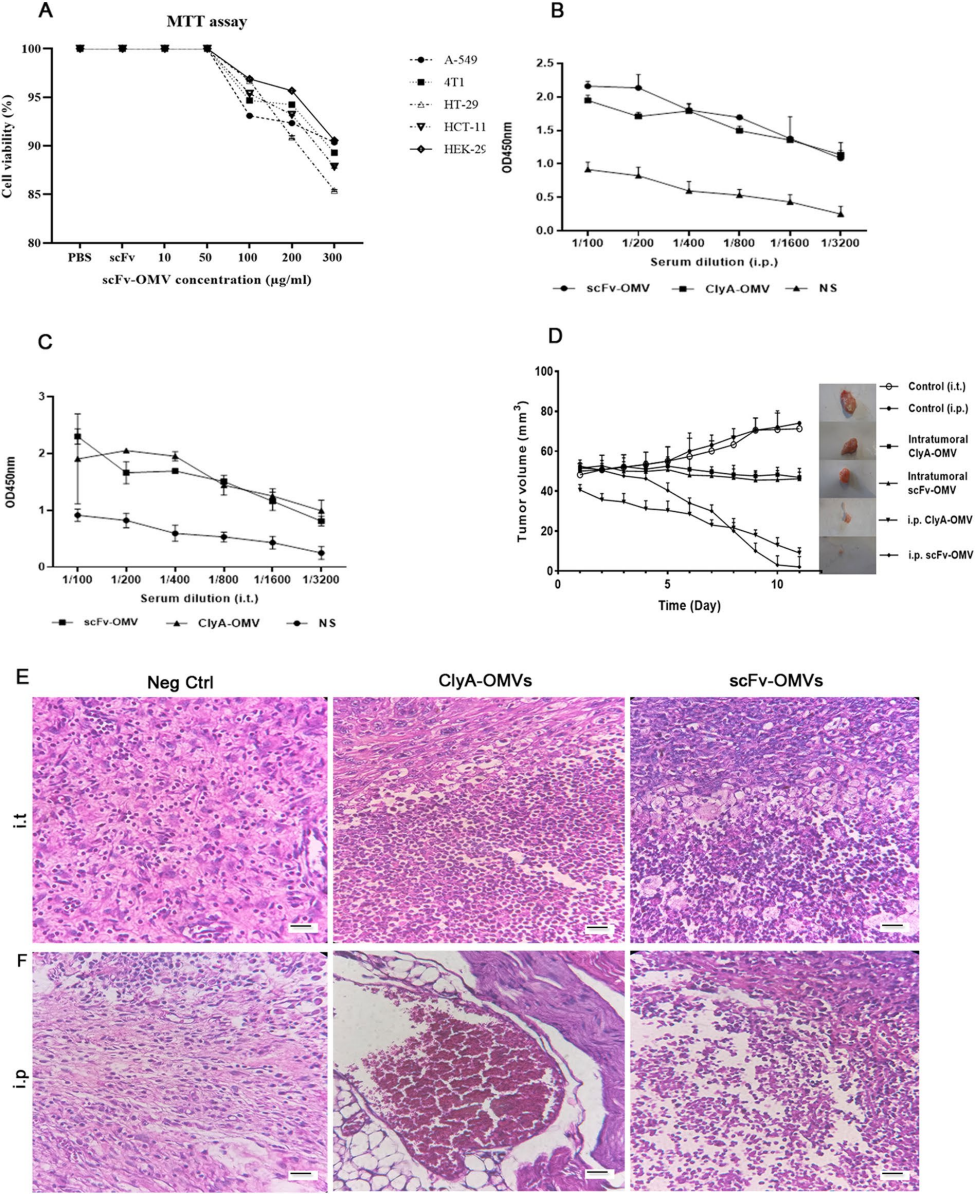
Anti-tumor efficiency evaluation of scFv-OMVs and ClyA-OMVs. (**A**) Cell viability assay performed 48 h post-treatment with scFv-OMVs. No significant differences were evident between cells treated with scFv-OMVs and negative control (PBS). All data displayed as mean ± SD (n = 3); (**B**) Serum polyclonal IgG titer in tumor-bearing mice subjected to i.p. administration of scFv-OMVs, ClyA-OMVs and normal saline (NS); (**C**) Serum polyclonal IgG antibody titers of TNBC mice models subjected to intratumoral injection of scFv-OMVs, ClyA-OMVs, and NS as negative control; (**D**) Average tumor volume across six groups; (E) H & E staining of tumor sections obtained from i.t. groups; (F) H & E staining of tumor tissues of mice treated intraperitoneally. Scale bar represent 100 µm. Data were represented as mean ± SD (n = 3). P < 0.05 comparing to control group.

The in vitro anti-tumor efficacy of scFv-OMVs was quantitatively assessed using the MTT assay. The results demonstrated that transfection with scFv-OMVs, ClyA-OMVs, and scFv did not cause any significant cytotoxic effects on Ht-29, HCT-116, A549, 4T1, and HEK-293T cell lines compared to the PBS-treated cells (Fig. 5A). However, cell viability was reduced to approximately 50% following LPS treatment, which served as the positive control. This finding confirms the safety of purified OMVs and indicates the absence of LPS-related toxicity in the experimental setup. The MTT assay results suggest that scFv-OMVs do not exhibit significant cytotoxic effects on the tested cell lines, indicating their potential safety as a delivery platform for therapeutic applications.

### scFv-OMVs induce immune activation in vivo

**Figure 6:**
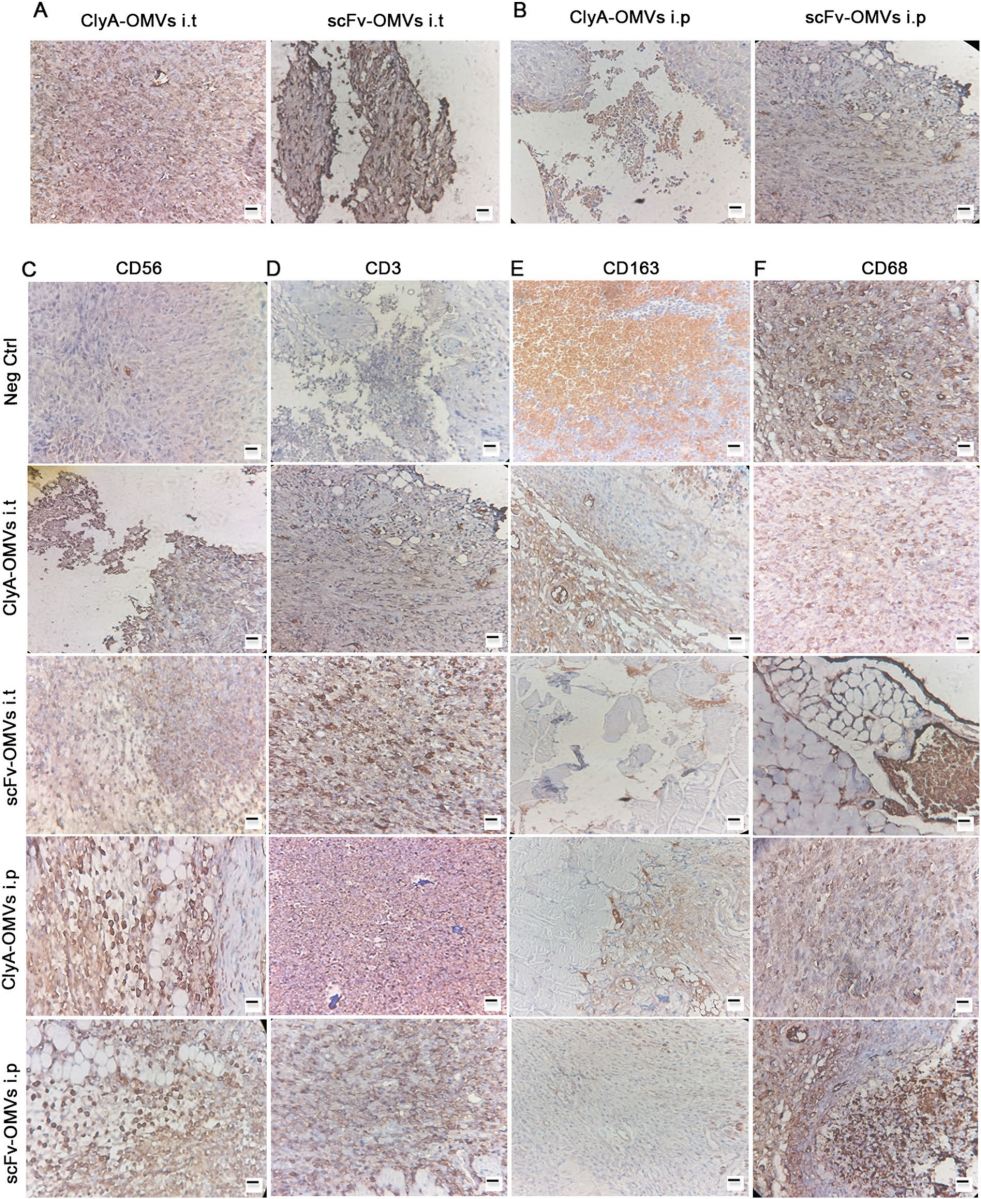
Immunohistochemistry (IHC) analysis of tumor tissue sections. (**A**) IHC staining using anti-His tag antibody aimed at detecting ClyA-OMVs and scFv-OMVs in tumor sections administered i.t.; (**B**) Similar IHC staining targeting ClyA-OMVs and scFv-OMVs within tumor tissue of mice treated intraperitoneally; (**C**) IHC staining of tumor sections with anti-CD56 antibody, serving to identify NK cells; (**D**) IHC staining utilizing anti-CD3 antibody facilitating the detection of T cell in tumor tissue; (**E**) IHC analysis using anti-CD163 antibody, in conjunction with; (**F**) anti-CD68, used for quantifying the M2/M ratio. This quantification offers insights into the impact of engineered OMVs on macrophage polarization within tumor tissue. Scale bar represents 100 µm.

**Figure 7:**
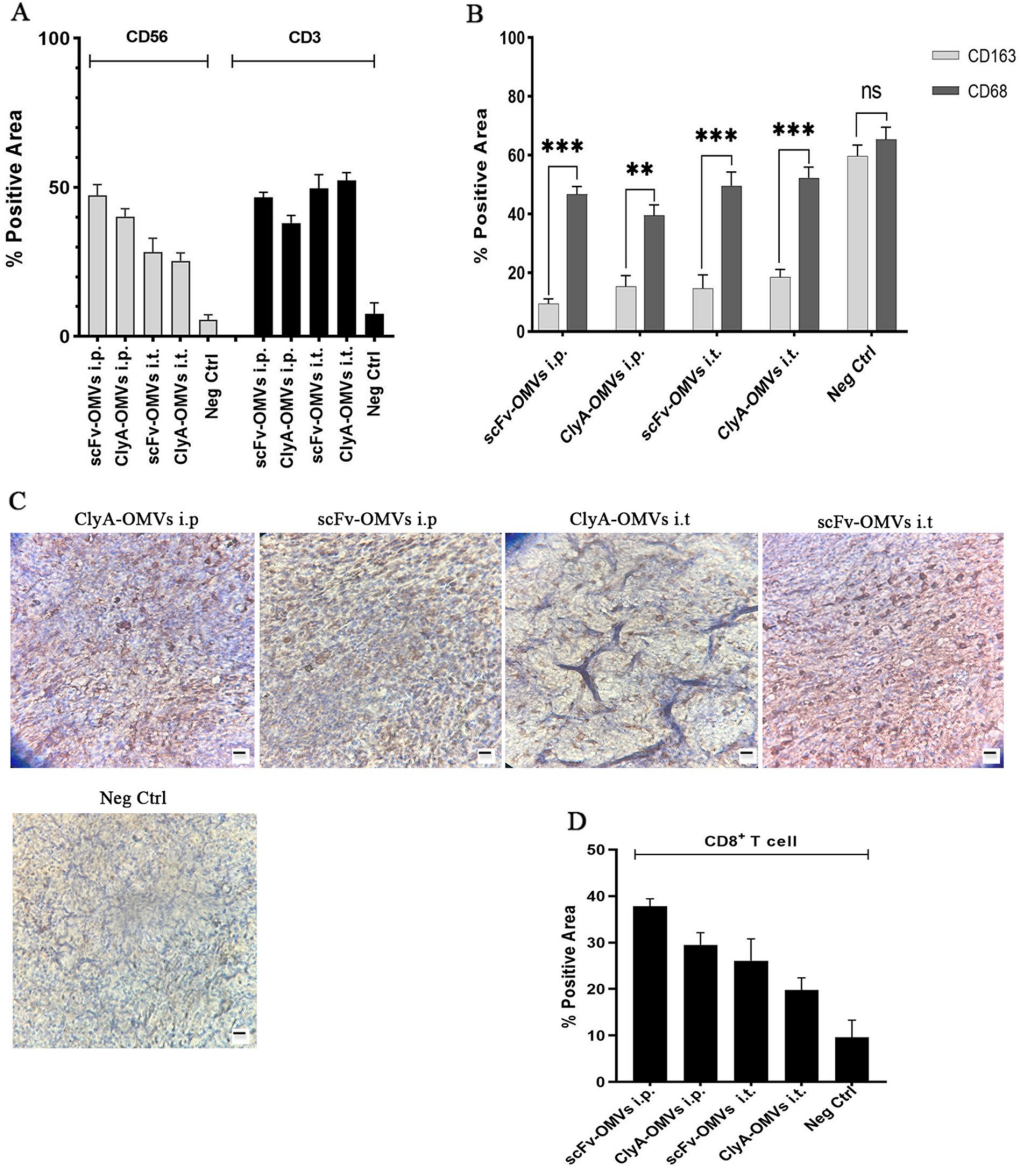
(**A**) The quantitative evaluation of immunopositive areas (ImageJ software) corresponding to NKs and T cells; (**B**) Determination of M2/M ratio; (**C**) IHC staining of tumor sections targeted at detecting CD8, a marker for CD8^+^ T cells. Scale bar is indicative of 100 µm; (**D**) The percentage of positive area concerning CD8^+^ T cells. Data were shown as mean ± SD (n = 3); statistical significance (*P < 0.01) as compared to the control group.

Subcutaneous injection of 4T1 cells into Balb/C mice resulted in the development of syngeneic transplanted breast cancer tumors. Tumor-bearing mice were then treated with ClyA-OMVs, scFv-OMVs, or PBS through intraperitoneal (i.p.) or intratumoral (injection directly into the tumor) administration for 11 consecutive days. Throughout the experiment, there were no significant fluctuations in the temperature and body weight of the mice, and no lethality was observed. These findings suggest that the administration of ClyA-OMVs and scFv-OMVs did not induce any noticeable adverse effects on the mice and that the treatments were well-tolerated. The results revealed a significant inhibition of tumor growth in mice treated intraperitoneally (i.p.) with both scFv-OMVs and ClyA-OMVs. This effect was observed throughout the treatment period of 11 days. In contrast, when the OMVs were administered directly into the tumor (intratumoral administration), there was no substantial alteration in tumor size, and the tumors appeared to remain relatively unchanged (Fig. 5). Despite the lack of visible changes in tumor size with intratumoral injection, histological analysis of tumor sections indicated different outcomes. In mice subjected to i.p. administration of both scFv-OMVs and ClyA-OMVs, a conspicuous occurrence of tumor necrosis was noted. In contrast, when ClyA-OMVs and scFv-OMVs were administrated via intratumoral injection, the induction of tumor necrosis was observed with a milder intensity. The culmination of in vivo experiments, coupled with the evaluation of immune cell infiltration percentage within the tumor tissue, conclusively highlighted the heightened anti-tumor efficiency of scFv-OMVs in comparison to ClyA-OMVs. This observation aligns with previous studies that have shown naturally derived OMVs to be effective stimulators of systemic anti-tumor immunity^3,14^. Naturally derived OMVs can carry various antigens, including pathogen-associated molecular patterns (PAMPs), which directly stimulate both innate and adaptive immunity^4,15–17^. When exposed to OMVs, antigen-presenting cells like macrophages M2 can absorb and process the PAMPs, leading to a pro-inflammatory response through cytokine secretion. This, in turn, triggers the maturation of dendritic cells (DCs), a crucial step in the activation of adaptive immune responses. To investigate whether OMVs can stimulate innate immunity, the presence of macrophages in the tumor tissue was verified through immunohistochemistry staining using the CD68 antibody. This analysis aims to confirm the recruitment of macrophages to the tumor site, potentially as a result of OMV treatment, which could contribute to the induction of an anti-tumor immune response. These findings suggest that scFv-OMVs may elicit a more robust and comprehensive immune response compared to ClyA-OMVs, which could explain their enhanced anti-tumor activity in in vivo experiments. Understanding the mechanisms behind the immune-stimulatory properties of OMVs is crucial for the development of effective cancer immunotherapies using these nanovesicles. The immunohistochemistry (IHC) results shown in Fig. 6 indicate a high level of CD68 marker expression in the tumor tissues of mice treated intraperitoneally (i.p.) and intratumorally with both ClyA-OMVs and scFv-OMVs. This high expression represents the accumulation of macrophages, particularly the M2 subtype, in the tumor microenvironment. However, staining with an anti-CD163 antibody confirmed a decrease in the level of M2 macrophages and a shift towards M1 macrophages compared to the control group. This shift is indicative of a change in the macrophage phenotype towards a pro-inflammatory state, which can be beneficial in anti-tumor immunity. In addition, staining with anti-CD3 and anti-CD56 markers provided substantiation for the presence of T cells and NK cells within the tumor tissue, respectively. This corroborates the active participation of both T cells and NK cells in tumor microenvironment. The coexistence of T cells (adaptive immunity) and NK cells (innate immunity) implies that the administration of OMVs triggered the activation of both innate and adaptive immune responses within the tumor microenvironment. This dual activation underscores the comprehensive impact of OMVs on various components of the immune system, fostering a multi-faceted anti-tumor effect. Upon comprehending the anti-tumor mechanisms orchestrated by OMVs, we embarked on an investigation into the role of M1 macrophages in the activation of CD8^+^ T cells within the tumor site. Notably, the outcomes derived from IHC analysis confirmed the constructive contribution of cytotoxic T cells (CTLs) to the anti-tumor response elicited by OMVs (Fig. 7). Collectively, the IHC results depicted in both Figs. 6 and 7 confirmed the capacity of OMVs, whether ClyA-OMVs or scFv-OMVs, to elicit a multi-faceted immune response within the tumor microenvironment. This intricate response encompasses the activation of macrophages, NK cells, and T cells. The concerted activity of these immune components synergistically contributes to the observed potential anti-tumor effects in the in vivo experiments. The notion of triggering humoral immunity against EGFR-targeted OMVs introduces a compelling proposition: anti-OMV antibodies may possess the capability to incite the antibody-dependent cellular toxicity (ADCC) response. This hypothesis finds support in the detection of serum IgG antibody attachment to OMVs through ELISA assay, as well as, the specific binding of scFv-OMVs to diverse EGFR-overexpressed cancer cells (Fig. 5B,C).

## Discussion

TLRs play a critical role in both innate and adaptive immunity and have emerged as promising targets in cancer therapy. Currently, only three TLR agonists have received FDA approval for use in patients^18^. In the tumor microenvironment (TME), TLRs are expressed by various immune cells, including monocytes, macrophages, dendritic cells, B cells, T cells, mast cells, and natural killer (NK) cells. Additionally, TLRs can be found on non-immune cells such as epithelial cells, fibroblasts, and even cancer cells^19,20^. TLR signaling in immune cells leads to anti-tumor response through immunostimulation and cytokine secretion that promote anti-tumor immunity. However, TLR activation in cancer cells can have the opposite effect, promoting tumor growth and progression^21^. To maximize the benefits of TLR activation in cancer therapy, it is essential to selectively target TLRs in the appropriate immune cells while avoiding their activation in tumor cells^22^. This targeted approach can enhance anti-tumor immunity and improve the therapeutic outcome. Most TLR agonists used as single therapies have not shown significant success in clinical trials due to low efficacy or severe side effects^23^. However, recent research has focused on combinatorial strategies in certain types of tumors, resulting in potent, safe, and prolonged anti-tumor responses^24–26^. OMVs secreted by bacteria carry various PAMPs, such as lipoproteins, flagellin monomers, and bacterial DNA fragments. The presence of TLR agonists on OMVs plays an important role in their ability to efficiently stimulate both innate and adaptive immunity^4^. The potential of OMVs as immunotherapy agents has been demonstrated in previous research^3,27–29^. While monotherapy with bacterial OMVs can be effective for some less destructive tumors such as CT26 and MC38 adenocarcinoma^3^, combination therapies with immune checkpoint inhibitors or other immunotherapy modalities are currently an area of intense research^30^. In this study, we designed OMVs carrying scFv structure originating from panitumumab monoclonal antibody to target EGFR overexpressing tumors. The in vitro results indicated a specific affinity of scFv-OMVs toward EGFR overexpressed cancer cells. Accordingly, the potential of designed OMVs in stimulating the immune system was investigated. Among various administration routes, local delivery by intratumoral administration (i.t.) has gained enthusiasm due to its low toxicity and efficacy in both primary tumors and distant metastases. However, systemic administration of advanced pharmaceutical formulations has shown the ability to limit drug clearance, reduce toxicity, enhance tumor accumulation, and improve the delivery of TLR agonists to the intended location. However, we also conducted a comparative analysis of systemic administration via the i.p. route and i.t. administration to elucidate the role of system-wide immune system activation in tumor eradication rather than attributing the reduction in tumor size solely to the properties of OMVs themselves. Furthermore, in line with the results of the MTT assay, the mode of action of OMVs featuring various surface proteins is anticipated to leverage anti-tumor immunity without directly inducing cytotoxic effects. Additionally, it is worth noting that immune-related adverse effects primarily stem from individual patient immune responses and susceptibility rather than the specific dosage or exposure levels of the drug. Therefore, the dosing strategies employed in this study are adopted from other studies^3,27,31^. The size of tumors in i.p. showed a significant decrease while in i.t. the size of tumors remained constant as compared to control groups. Analysis of the immunohistochemistry (IHC) results using the anti-His tag antibody confirmed the delivery of our OMVs into tumor tissue through the i.p. route. Furthermore, the presence of natural killer (NK) cells and T cells in tumor tissue was examined in all groups. Remarkably, both i.p. and i.t. administration of ClyA-OMVs and scFv-OMVs prompted the aggregation of these vital immune cells within the tumor microenvironment. In alignment with this, our investigation extended to analyzing the ratio of M2 to total macrophage cells within the tumor tissue. This scrutiny was aimed at unraveling the intricate role of engineered OMVs in fostering macrophage polarization via TLR agonists embedded within the OMVs architecture. The finding yielded affirmative outcomes, signifying a notable reduction in the presence of M2 macrophages coupled with a shift towards the M1 phenotype in the tumor nest. This transformation in macrophage polarization along with comparison of M2 to total macrophage in tumor nest and stroma lent additional support to the constructive impact of OMVs on inhibiting tumor growth. The presence of M1 macrophages in the tumor microenvironment is associated with a pro-inflammatory response, which may contribute to the suppression of tumor growth M1 macrophages are notably responsive to stimuli like LPS/IFNγ and hold significance as prolific producers of pro-inflammatory cytokines. Previous investigations have explored the role of OMVs in stimulating IFNγ production within the tumor tissue^3^. Notably, the TLR agonists on the surface of OMVs substantially contribute to the macrophage polarization process. The presence of M1 macrophage aligned with the concurrent infiltration of cytotoxic CD8^+^ T cells (CTLs), recognized as potent effectors in anti-cancer immune response (Fig. 7) highlights a closely interlinked orchestration of immune mechanisms. Additionally, the evaluation the positive area percentage, particularly pertaining to i.p. administration of both scFv-OMVs and ClyA-OMVs, corroborated a heightened accumulation of scFv-OMVs at tumor site as compared to ClyA-OMVs. This observation underscores the enhanced tumor-targeting capacity of scFv-OMVs an attribute that bolsters their potential therapeutic efficiency. This observation substantiates the pivotal role of scFv component in mitigating the tissue clearance of OMVs. By facilitating high affinity attachment to the surface of tumor cells, the scFv element enhances the OMVs retention within the tumor microenvironment. This enhanced accumulation may contribute to the improved anti-tumor response observed with scFv-OMVs. In concert, these outcomes collectively suggest that scFv-OMVs exert a pivotal influence on fostering the macrophage polarization, concurrently with the influx of cytotoxic CD8 + T cells within the tumor microenvironment. This effect combined with the enhanced propensity of scFv-OMVs to accumulate at tumor site lends robust credence to their potential as effective immunotherapeutic agents targeting tumor growth.

In conclusion, we present engineered OMVs targeting EGFR originating from *E. coli* species as immunotherapeutic agents, distinct from traditional vaccines or drug delivery systems, for triple-negative breast tumor cells. The in vitro results demonstrated that scFv-OMVs effectively targeted EGFR on the surface of various EGFR high-expressed cancer cells, indicating their potential effectiveness in different types of cancer models. The in vivo studies also support the potential application of OMVs as immunotherapy agents. Comparing the two different routes of administration highlighted the hidden role of other immune system components in the OMVs-mediated anti-tumor response. Collectively, these engineered nano-size vesicles represent a promising potential anti-tumor strategy for future investigations.

## Methods

All methodology applied in the present study was developed in accordance with national and international standards for the care and use of laboratory animals, with approval from the ethics committee in the use of animals at Shahed University (IR.SHAHED.REC.1399.094).

All experiments reported in this manuscript are in accordance with ARRIVE guidelines.

### Plasmid construction

The chimeric gene, consisting of a fusion of ClyA to scFv with a His tag and a flexible linker (SSSSGSSSSG), was synthesized by Biomatic Company (Canada) and cloned into the pET26b cloning vector. The scFv construct was PCR amplified using scFv forward (5′-CATGCCATGGGTCAAGTTCAGCTGCAA-3′) and reverse (5′-CGCGGATCCTTAGTGGTGATGGTGAT-3′) primers. The amplified sequence was then inserted between the *Bam H1* and *Nco1* sites of the pET28a. After digestion with *Bam H1* and isolation of scFv-His tag, the ClyA anchor in the pET26b vector was self-ligated using T4 DNA ligase to be used as a negative control throughout the study. The ligation products were transformed into *E. coli* BL21, DE3 and cultured on an LB agar plate containing 50 µg mL^−1^ kanamycin. Colonies were analyzed by *Bam H1* and *Nco1* for the presence of scFv structure and by *BamH1, Hind III* for ClyA. Positive clones were sequenced and purified for subsequent steps in the study. The recombinant scFv clones were cultured in 200 ml of LB broth medium with 50 µg mL^−1^ kanamycin at 37 °C until they reached an optical density at 600 nm (OD_600_) of 0.5–0.6. Following induction with 0.5 M isopropyl β-D-1-thiogalactopyranoside (IPTG), the culture was further incubated at 22 °C overnight. The cells were then collected by centrifugation at 5000 rpm for 10 min. The resulting pellet was resuspended in phosphate buffer (100 mM NaH_2_PO_4_, 10 mM Tris–HCl) and lysed by ultrasonication. The scFv protein was subsequently purified using the Ni–NTA column (Qiagen, Netherlands) according to the manufacturer’s instructions. Briefly, after equilibrating the column with binding buffer, the sample was loaded onto the column. Next, to refold the scFv protein, the Ni–NTA column was washed successively with 8 M, 4 M, and 2 M Urea in 100 mM NaH_2_PO_4_ and 100 mM Tris, and then with base buffer. To remove nonspecific binders, the column was further washed with 10 mM and 20 mM of Imidazole. Finally, the bound protein was eluted from the column using 500 mM Imidazole and its buffer was exchanged with PBS through dialysis. To assess the quality and identity of the purified scFv recombinant protein (49 kDa), SDS-PAGE analysis was performed. Additionally, an immunoblotting assay was carried out to confirm the presence of scFv protein. These analyses ensured the successful purification and proper folding of the scFv protein for further experiments.

### OMV isolation and purification

OMVs were prepared following the previously described method^5^. Briefly, the *E. coli* BL21 (DE3) strain transformed with a pET-26b ClyA-scFv construct was grown in 1000 ml LB broth batch culture at 37 °C till it reached an OD_600_ of 0.5–0.6. Protein expression was induced by adding 0.5 M IPTG, and the culture was then incubated at 22 °C overnight. The bacteria were harvested, and the cell pellets were treated with 7.5 times their wet weight of 0.1 M Tris-10 mM EDTA buffer. Extraction of the budding vesicles was carried out using 1/20^th^ volume of 0.1 M Tris–HCl (pH 8.6) buffer containing 10 mM EDTA and 0.5% (w/v) deoxycholate (Sigma-Aldrich, USA). The cellular debris was removed by centrifugation at 18,000 × g for 90 min at 4 °C. The vesicles in the supernatant were concentrated by ultracentrifugation (Beckman SW32Ti rotor) at 125,000 × g for 2 h at 4 °C. The resulting pellet containing OMVs was washed with 0.1 M Tris, 10 mM EDTA, and 0.5% (w/v) deoxycholate and then precipitated by centrifugation. Subsequently, the pellet of vesicles was suspended in 3% sucrose solution as a preservative, filtered through a 0.45 µm pore-size Millipore filter, and stored at 4 °C until used. The sterility of purified engineered OMVs was confirmed by the absence of bacterial colonies in agar plates incubated overnight at 37 °C. The total protein concentration of scFv-OMV and ClyA-OMVs was estimated using the modified Lowry method. The expression of the exogenous protein was confirmed by SDS-PAGE electrophoresis and immunoblotting technique.

### Characterization of the engineered OMVs

ClyA-OMV and scFv-OMVs were subjected to characterization in terms of morphology and size using transmission electron microscopy (TEM) and Zetasizer Nano-ZS apparatus on dynamic light scattering (DLS) techniques. For TEM analysis, samples were adsorbed on formvar carbon-coated grids for 2 min and negatively stained with 2% uranyl acetate. Subsequently, the grids were dried at room temperature. The Grids were then viewed under a Zeiss-EM10C electron microscope operating at an 80-kV accelerating voltage. The different types of vesicles were analyzed for their homogeneity, and the size of the vesicles was estimated using the analysis program provided by the Soft Imagin system in Switzerland. The size distribution and polydispersity index (PDI) of ClyA-OMV and scFv-OMV at the final concentration of 50 μg/ml in PBS were determined by the DLS technique. The Z-average size represents the mean diameter (d.nm) of the vesicles, while the polydispersity index (PDI) indicates the particle size distribution, reflecting their homogeneity or heterogeneity.

### Monitoring specificity of scFv-OMV for EGFR by cell-ELISA

To investigate the specificity of engineered OMVs for EGFR overexpressed cell lines (HCT-116, HT-29, 4T1, A549) and a negative control cell line (HEK-293T), an enzyme-linked immunosorbent assay (ELISA) was conducted. All cell lines were cultured in a DMEM medium containing 10% FBS. Subsequently, 7 × 10^3^ cells were seeded into each well of a 96-well plate (SPL, Korea) and incubated at 37 °C with 5% CO_2_ until they reached 70% confluency. Upon fixation with 100 µL formaldehyde 10%, each well was blocked with 200 µL 5% w/v skim milk in PBS. After washing with PBS containing 0.1% Tween-20 (PBST), all wells were coated with 100 µL of the scFv-OMV and ClyA-OMV at 10 µg per well, as well as scFv (5 µg per well) and then incubated at 25 °C for 2 h. Next, the scFv-OMVs were detected using 100 µL of HRP conjugated anti-His tag antibody (1/6,000 in PBST), followed by three washes with PBST. The immunoreactivity was visualized using the chromogenic HRP substrate 3,3′,5,5′-tetramethylbenzidine (TMB) from Abcam, United Kingdom), and the absorbance was measured at 450 nm to assess the binding of OMVs to the respective cell lines.

### Cell binding assessment by flow cytometry

The cell specificity and binding capacity of the scFv-OMV were assessed using the flow cytometry technique, following the method described by Zarei et al.^32^. In brief, 4 × 10^5^ cells of each of the cell lines (HCT-116, HT-29, A549, and HEK-293T) were centrifuged at 1800 × g for 5 min. The cells were then washed with FACS buffer (PBS, 5% FBS), and incubated with 25 µg of ClyA-OMV and scFv-OMV or 4 µg of scFv for 1 h at 4 °C. After two washes with FACS buffer, 100 µL of the anti-His tag antibody (1/100) was added to each microtube and incubated for 2 h at 4 °C. Then, cells were stained with FITC-conjugated secondary antibody (1/1000) in the dark for 20 min at 4 °C. After resuspending the cells in PBS, the fluorescent intensity of each sample was measured by flow cytometry. All samples were compared to a negative control with the same treatment except for OMV or scFv incubation. This analysis allowed the determination of the binding capacity of scFv-OMV to the respective cell lines in comparison to the negative control.

### Calculation of Kd by flow cytometry

The HCT-116 cell line with high EGFR density^33^ was used to determine the binding affinity of the designed scFv-OMV against EGFR. For estimation of Kd, different concentrations of scFv-OMVs, ranging from 0 to 80 µg mL^−1^ with approximately 0–6.5 µM scFv protein in total were incubated with 4 × 10^5^ of HCT-116 cells and subjected to flow cytometry as described above. The fluorescent intensity was then measured and the non-linear Kd regression curve was plotted using Sigma Plot 12.0 software and the inverse relationship. This curve provides valuable insights into the binding affinity and specificity of the scFv-OMVs to EGFR on the cell surface.

### Fluorescent microscopy

To visualize the in vitro cell binding of scFv-OMV, fluorescent microscopy (Olympus, BX61 model, Japan) was applied. For this purpose, we used EGFR-overexpressed A-549 cells and EGFR-negative HEK-293T cells. To determine the specific binding of scFv-OMV to EGFR, formaldehyde-fixed EGFR-overexpressed and EGFR-negative cells were incubated with scFv-OMV for 1 h at 4 °C, following cell binding assay protocol. The cell surface binding of scFv-OMV was then monitored by fluorescent microscopy. Subsequently, cells were exposed to bovine serum albumin 2% (w/v in PBS) for 1 h to block non-specific binding. After washing, anti-His tag primary antibody and FITC-conjugated anti-mouse secondary antibody were added to each well, respectively. To stain the nuclei, propidium iodide (PI) at a concentration of 1 µg/ml was applied (Sigma-Aldrich, United States).

### Cell viability assay (MTT)

The cytotoxic effect of scFv-OMV was quantitatively assessed using the colorimetric, tetrazolium-based MTT assay. Four EGFR-overexpressing cell lines, namely HCT-116, HT-29, 4T1, and A549, along with the HEK-293T cells as a control, were treated with different agents for 48 h. The treatments included PBS, scFv-OMV, ClyA-OMV (nontargeted OMV), and scFv protein. After the treatment period, the medium was removed, and 30 µL of MTT solution (2.5 mg/ml) was added to each well. The cells were then incubated for 4 h at 37 °C to allow the formation of the formazan product. Next, the MTT solution was carefully removed, and 100 µL of dimethyl sulfoxide (DMSO) was added to dissolve the formazan crystals in each well. The absorbance of the resulting solution was measured at 570 nm using a microplate reader (Multiskan Go, Thermo Scientific), and the cell viability was calculated as the percentage of viable cells in the treated groups relative to the control group. All data presented in this study are representative of mean values of three readings and P value < 0.05 were considered statistically significant.

### Antitumor experiment

Antitumor studies were performed using 4–6-week-old female BALB/c mice (Royan Institute of Iran) as described previously^31^. Animals were kept in a specific pathogen-free environment following 12 h-day–night cycle, randomly divided into about 3 mice per cage. Animals were housed under standard conditions, with access to food and water ad libitum, and cared for by trained staff. To establish the 4T1 syngeneic tumor mice models, 1 × 10^6^ cells suspended in 100 µL of serum-free medium in their exponential growth phase were subcutaneously injected into the flank of the hind leg of mice. After 10 to 12 days, when the tumor diameter reached around 0.7 mm, the mice were randomly divided into 6 groups for sample treatment. Mice were treated daily with intratumoral injection of 10 µg/dose of ClyA-OMV, scFv-OMV, and normal saline (NS) as control or intraperitoneally with 25 µg/dose of ClyA-OMV, scFv-OMV, and NS. The study design allowed for a blind randomization to minimize bias in the evaluation of treatment outcomes. Tumor volume (mm^3^) was calculated as (Length × Width^2^)/2. Eleven days later, the mice were subjected to dissection, and tumors were removed for further analysis. Blood samples were also collected for ELISA assay and other biochemical detection.

### Tumor tissue immunohistochemistry

Tumor tissues were fixed with a 10% formaldehyde solution for three days and then embedded in paraffin blocks. Then, paraffin blocks were sectioned to 4 µm thickness and were deparaffinized with xylene and rehydrated by decreasing the ethanol concentration before further analysis. The sections were stained with hematoxylin and eosin (H&E) for histological study and then observed by inverted microscopy (Nikon, Japan). For immunohistochemistry analysis, on deparaffinized sections were treated with 3% hydrogen peroxide for 30 min to block endogenous peroxidase. Then the sections were incubated with primary antibody against CD56 for NK cells, CD3 for T cells, CD68, and CD163 at 4 °C overnight for detection of macrophage M2 and CD8 for detection of CTL cells. In the next step, sections were treated with a secondary antibody for 1.5 h at room temperature. Following PBS washing, sections were exposed to 3,30 -diaminobenzidine (DAB) chromogenic solution for 10 min, and then washed with PBS and counterstaining with hematoxylin. To ensure the specificity of the antibody, a negative control without the primary antibody was included. All sections were visualized with inverted microscopy (Nikon, Japan), and the percentage of the positively stained surface to the total surface was determined and quantified by ImageJ software (https://imagej.net/ij/index.html). Then the CD163/CD68 ratio was defined as the ratio between the CD163-positive stained cells and CD68-positive stained cells in tumor tissues. This analysis allowed us to assess the presence and distribution of specific immune cells in the tumor microenvironment.

### Serum antibody analysis by indirect ELISA

The level of polyclonal IgG isotype to OMVs was determined by ELISA using a 96-well ELISA microplate (MaxiSorb; Nunc, Wiesbaden, Germany). Each well was coated with 1 µg of OMVs in carbonate/bicarbonate buffer and blocked with 5% (w/v) skim milk in PBS for 2 h and then washed with PBS. Serial dilutions of serum obtained from all treated groups were added to wells and incubated for 2 h at 37 °C. After washing, the anti-mouse antibody conjugated with HRP (Biolegend, San Diego, USA) was added to the wells and incubated for 2 h at 37 °C. The chromogenic HRP substrate TMB (Abcam, United Kingdom) was added and the reaction was stopped by 3N sulfuric acid, then the absorbance was measured at 450 nm. The absorbance was measured at 450 nm to determine the levels of polyclonal IgG isotype to OMVs in each sample. This analysis allowed us to evaluate the humoral immune response and the production of specific antibodies against the OMVs. The data presented throughout the study are mean values obtained from three separate readings and P value < 0.05 were statistically considered significant.

### Statistical analysis

All presented data from the ELISA assay and flow cytometry assay are the mean values of three readings. Statistical comparisons between different groups were executed One-way ANOVA analysis. Differences with a P value < 0.05 were considered significant. For the statistical analysis, GraphPad Prism software, version 6, and IBM SPSS software, version 22 were used.

### Supplementary Information

Supplementary Figures.

## Data Availability

All data generated or analyzed during this study are included in this published article.

## Abbreviations

TLR: Toll-like receptor
APC: Antigen presenting cells
OMVs: Outer membrane vesicles
EGFR: Epidermal growth factor receptor
i.p.: Intraperitoneal
i.t.: Intratumoral
scFv: Single-chain fragment variable
ClyA: Cytolysin A
NK: Natural killer cells
ADCC: Antibody-dependent cellular cytotoxicity
PAMPs: Pathogen-associated molecular patterns
ELISA: Enzyme-linked immunosorbent assay
IHC: Immunohistochemistry
H&E: Hematoxylin and eosin
HRP: Horseradish peroxidase
Kd: Equilibrium dissociation constant
PDI: Polydispersity index
PI: Propidium iodide
MTT: Cell viability assay
