# Supplementary material for: Anti-EGFR bioengineered bacterial outer membrane vesicles as targeted immunotherapy candidate in triple-negative breast tumor murine model
Source: Sci Rep. 2023 Sep 29;13:16403. doi: 10.1038/s41598-023-43762-y (PMC10541432; doi:10.1038/s41598-023-43762-y)
Supplement: Supplementary file 1 — Supplementary Figures. [file 41598_2023_43762_MOESM1_ESM.pdf]

Anti-EGFR Bioengineered Bacterial Outer Membrane Vesicles as Targeted Immunotherapy Candidate in Triple-Negative Breast Tumor Murine Model

Razieh Rezaei Adriani[1], Seyed Latif Mousavi Gargari[1,*], Hamid Bakherad[2], Jafar Amani[3]

Supplementary File

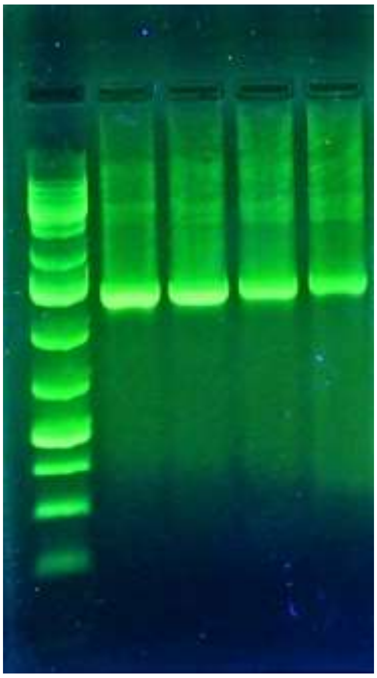

FigS1. The PCR results for the scFv structure obtained using a distinct set of forward and reverse primers across four varying annealing temperatures.

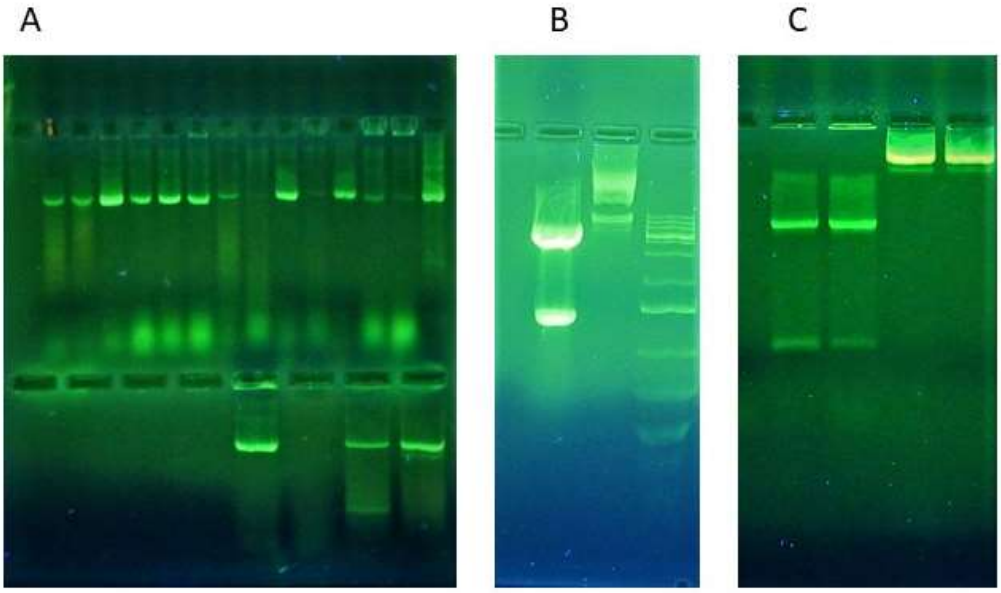

Fig.S2 (A) The results of colony PCR performed on transformed strains using scFv-specific primers after acquiring the scFv-pET28a construct; (B) The electrophoretic patterns of the extracted scFv structure from the selected recombinant colonies post-digestion with *NcoI* and *BamH1* restriction enzymes; (C) Validates the presence of the recombinant colonies containing the ClyA-pET26a construct through *HindIII/BamH1* restriction enzyme digestion, visualized on a 1% agarose gel.

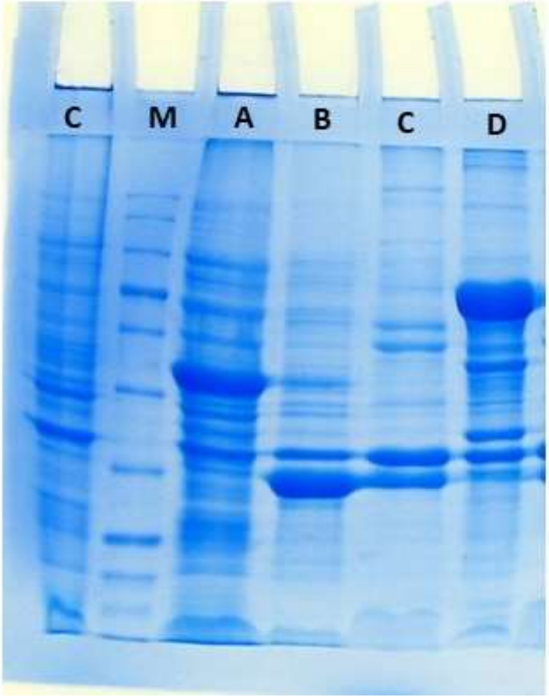

Fig.S3 Protein analysis of recombinant proteins on 12% SDS-PAGE. (C) Control: the protein content of the *E. coli* BL21 lacking vector; (M) protein marker; (A) protein content of *E. coli* BL21 containing recombinant scFv; (B) recombinant ClyA; (C) OMVs carrying recombinant ClyA; (D) protein content of *E. coli* BL21 containing recombinant ClyA-scFv fusion protein**.**

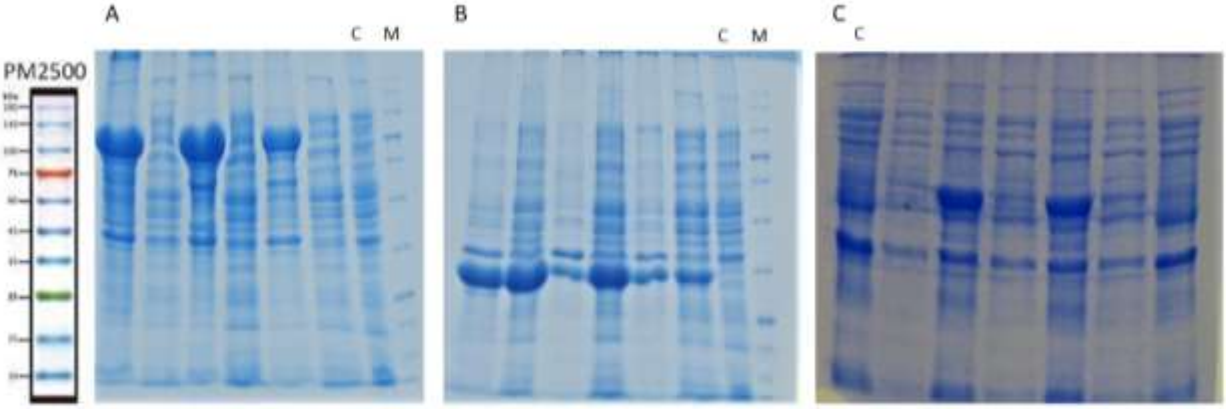

Fig.S4 (A) Optimization of ClyA-scFv expression across varying temperatures; (B) ClyA expression under different temperature conditions; (C) scFv expression at different temperature settings.

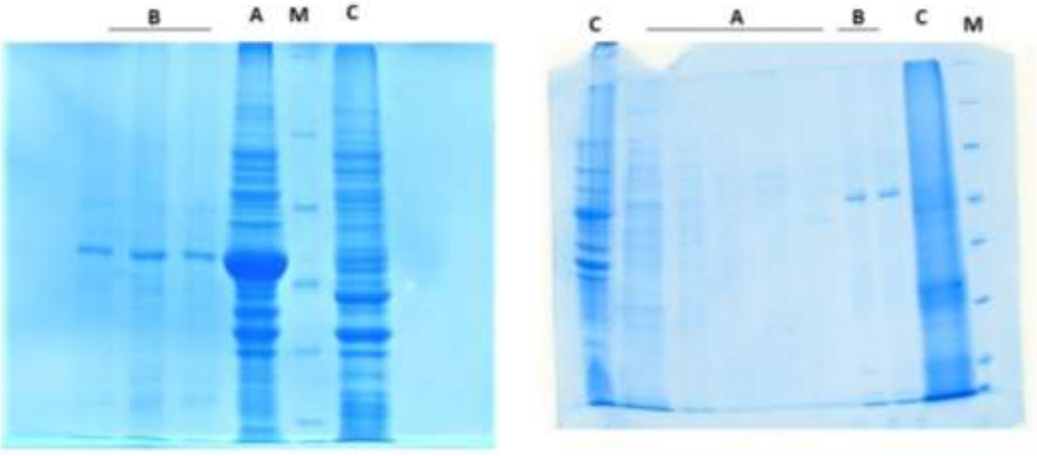

Fig.S5 (A) Depict the scFv purification using a Ni-NTA column, C: Negative control, M: Protein Marker, A: positive control, B: purified scFv; (B) C: Negative control, A: Elution from the column after successive washes with 8 M, 4 M, and 2 M Urea in 100 mM NaH_2PO_4, along with 10 mM and 20 mM of Imidazole, B: Elution of scFv protein following a wash with 500 mM Imidazole, C: washing the column with NaOH

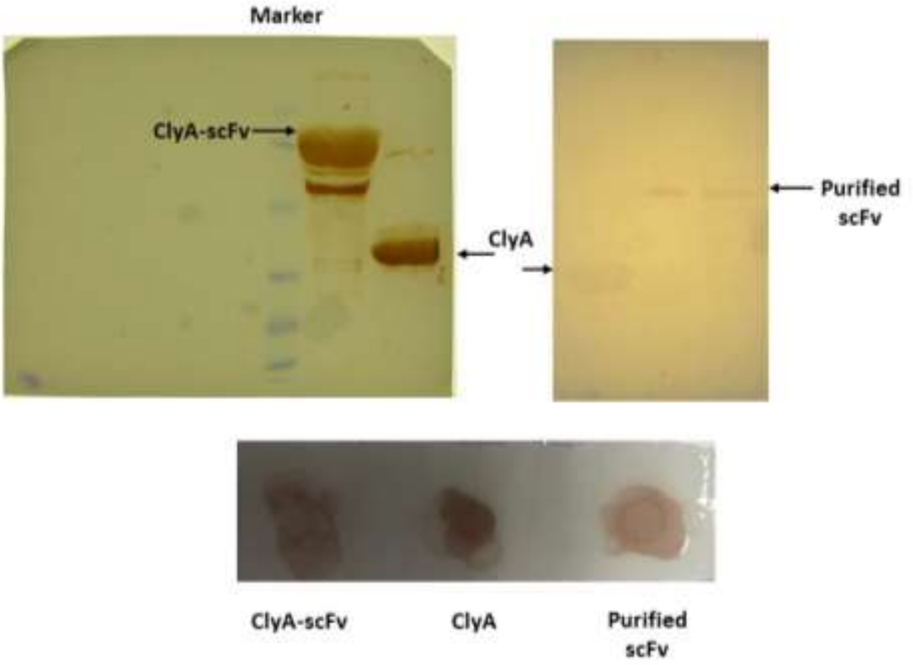

Fig.S6 (A) Western blotting conducted on total bacterial protein samples; (B) Western blotting of purified scFv protein, (C) Dot blot analysis of three recombinant proteins.

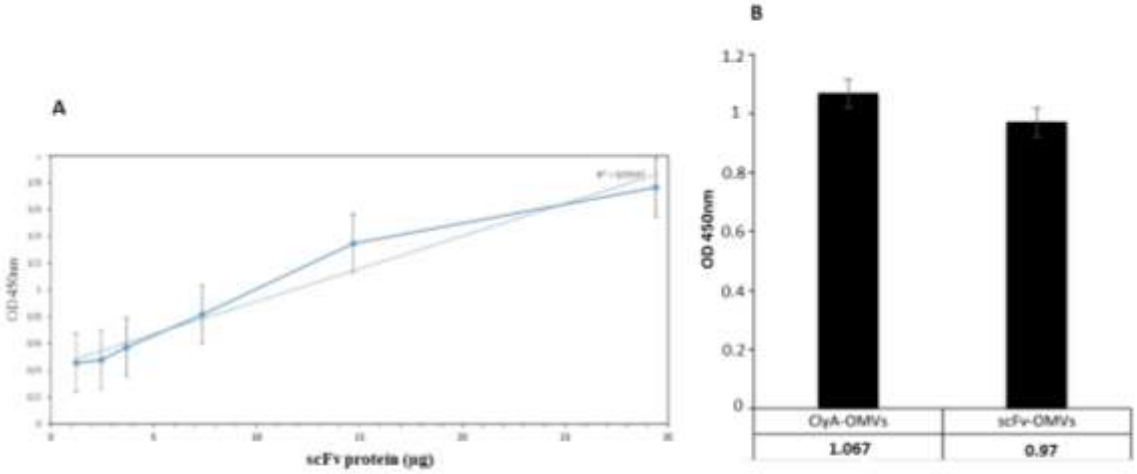

Fig.S7 Protein assay using an ELISA test to ascertain the extent of ClyA and scFv presentation on the surface of OMVs. (A) the standard curve involving the purified scFv protein at different concentrations within the ELISA assay; (B) the ELISA results for ClyA-OMVs and scFv-OMVs.
